# Supplementary material for: The psychological impact of major disasters on Japan’s medical system: An SNS text analysis
Source: PLoS One. 2026 Feb 20;21(2):e0343019. doi: 10.1371/journal.pone.0343019 (PMC12923033; doi:10.1371/journal.pone.0343019)
Supplement: S1 Text — These words were designated for forced extraction to rectify inappropriate segmentation patterns identified during the preliminary analysis using KH Coder. (PDF) [file pone.0343019.s001.pdf]

S2 Appendix. Pretreatment for KH Coder analysis

Settings for forced extraction words.

お薬手帳	**Medicine record book**
マイナ	**My number**
マイナンバー	**My number**
ポータル	**Portal**
スマホ	**Smartphone**
