# Supplementary material for: The psychological impact of major disasters on Japan’s medical system: An SNS text analysis
Source: PLoS One. 2026 Feb 20;21(2):e0343019. doi: 10.1371/journal.pone.0343019 (PMC12923033; doi:10.1371/journal.pone.0343019)
Supplement: S1 Table — The dataset was randomly extracted using a Python script. Two authors independently determined the ground truth labels for the 400 posts. Disagreements were initially addressed through discussion; however, in cases where a consensus could not be reached, both sentiments were adopted as the final ground truth. (PDF) [file pone.0343019.s002.pdf]

	Both positive	Human only	ML-Ask only	Both negative	Kappa
Anger	18	8	11	363	0.629
Dislike	21	12	12	355	0.604
Excitement	4	5	7	384	0.385
Fear	43	11	24	322	0.660
Fondness	1	11	3	385	0.112
Gloom	6	6	9	379	0.425
Joy	4	10	6	380	0.313
Relief	7	7	9	377	0.446
Shame	9	2	13	376	0.660
Surprise	4	5	7	384	0.385
				Average	0.449
