# Supplementary figures and images for: The psychological impact of major disasters on Japan’s medical system: An SNS text analysis
Source: PLoS One. 2026 Feb 20;21(2):e0343019. doi: 10.1371/journal.pone.0343019 (PMC12923033; doi:10.1371/journal.pone.0343019)

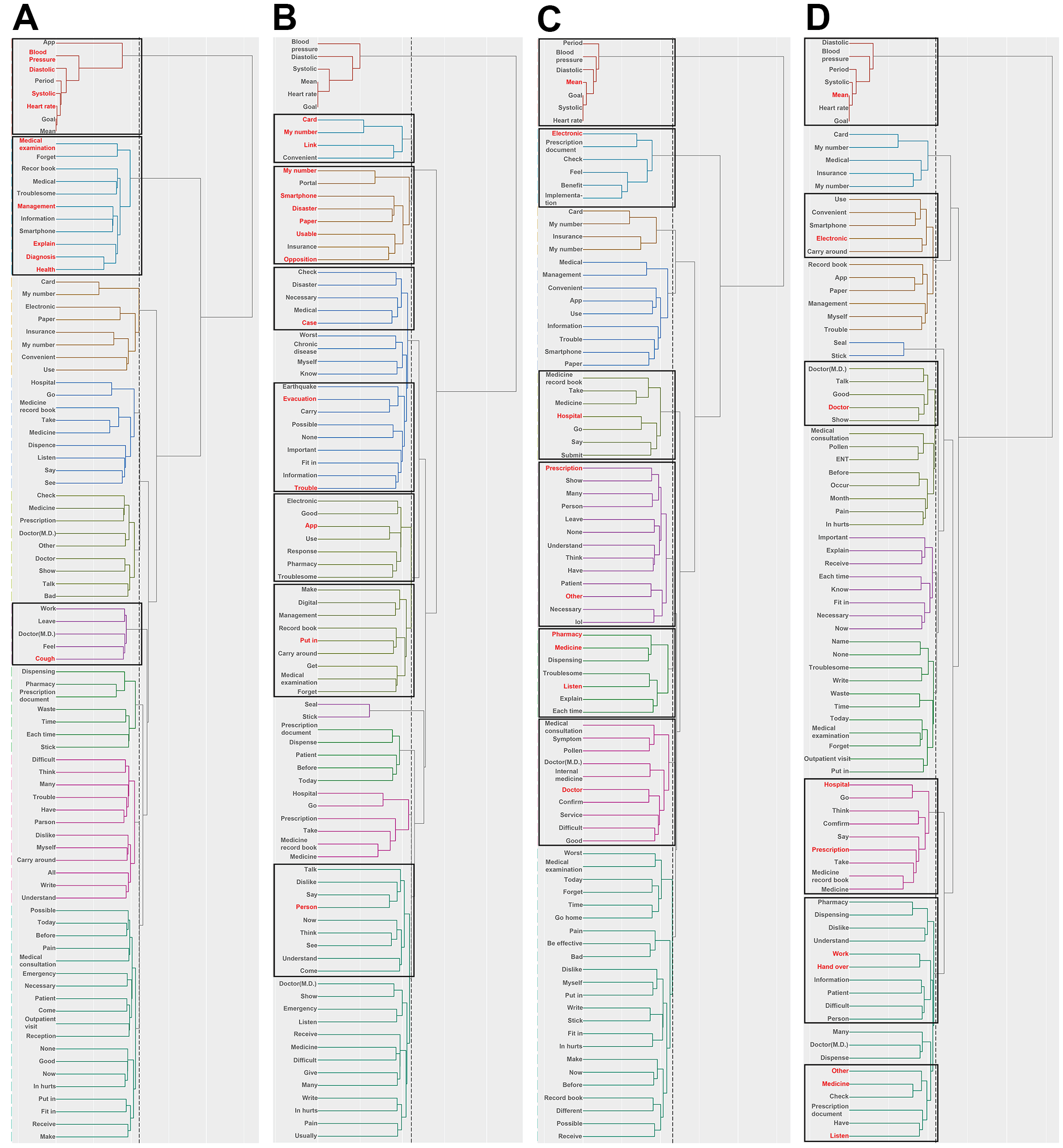

Supplement: S1 Fig — All analysis results are shown. The black boxes highlight areas of strong correlation in the correspondence analysis of posts related to anger. Each figure indicates the results for the following periods: A: October to December, B: January, C: February and D: March. (TIF) [file pone.0343019.s003.tif]

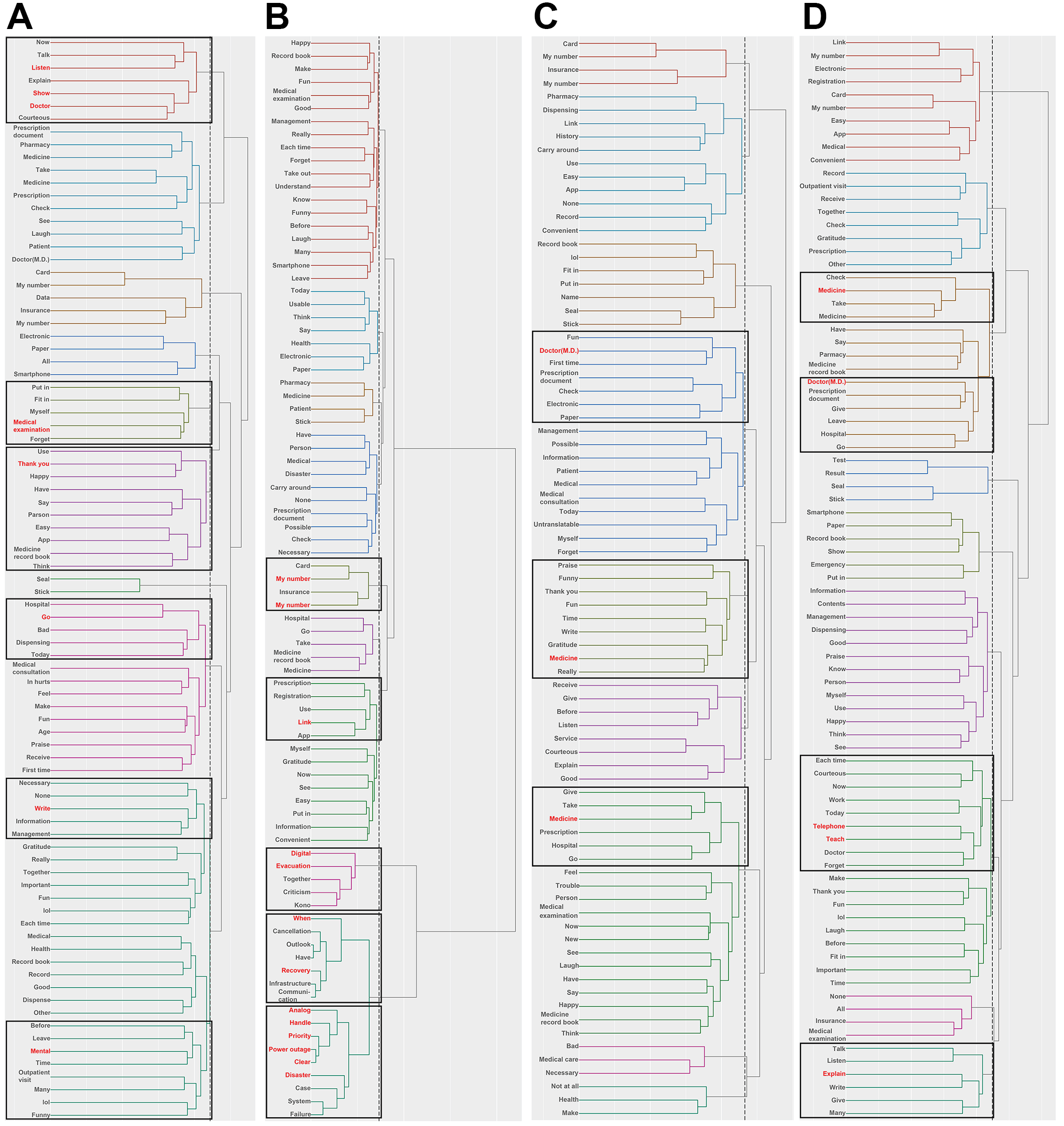

Supplement: S2 Fig — All analysis results are shown. The black boxes highlight areas of strong correlation in the correspondence analysis of posts related to anger. Each figure indicates the results for the following periods: A: October to December, B: January, C: February and D: March. (TIF) [file pone.0343019.s004.tif]

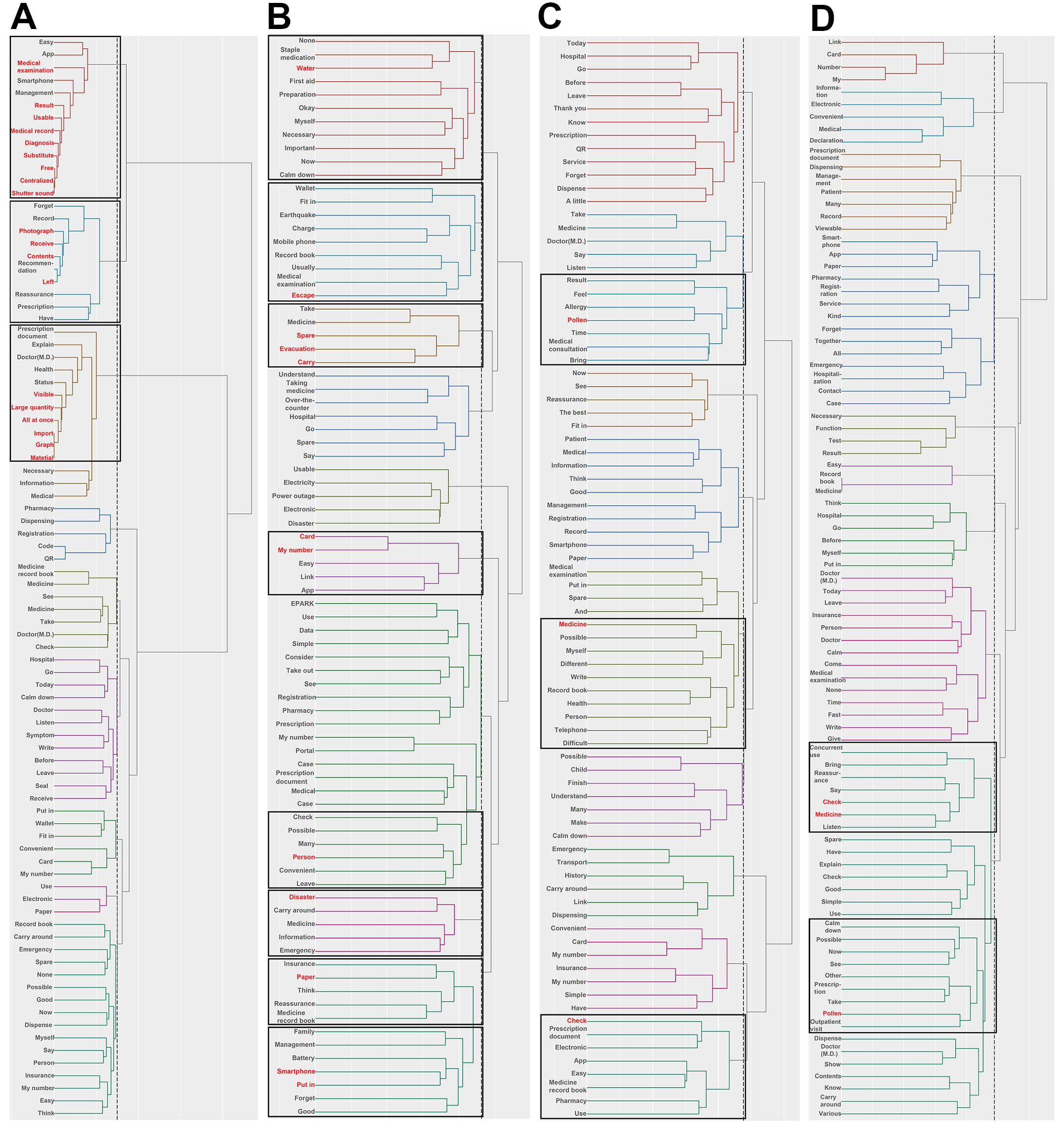

Supplement: S3 Fig — All analysis results are shown. The black boxes highlight areas of strong correlation in the correspondence analysis of posts related to anger. Each figure indicates the results for the following periods: A: October to December, B: January, C: February and D: March. (TIF) [file pone.0343019.s005.tif]
